# Managing water on plant leaf surfaces: roles and regulation of hydrophobicity

**DOI:** 10.1093/jxb/erag112

**Published:** 2026-02-27

**Authors:** Shumpei Hashimoto, Jun-Ichi Itoh

**Affiliations:** Graduate School of Agricultural and Life Sciences, The University of Tokyo, Tokyo 113-8657, Japan; Graduate School of Agricultural and Life Sciences, The University of Tokyo, Tokyo 113-8657, Japan; Nagoya University, Japan

**Keywords:** Adaptation, epicuticular wax, epidermal structure, genetic regulation, hydrophobicity, papilla, superhydrophobicity

## Abstract

Hydrophobicity—the ability of plant surfaces to repel water—is a fundamental physical property that governs interactions between plants and their environment. It plays vital roles in pathogen defense, photosynthetic efficiency, and water balance. Although long recognized as a defining feature of terrestrial plants, the mechanisms underlying the formation and regulation of surface hydrophobicity have only recently begun to be elucidated at the structural and genetic levels. This review integrates current understanding of the biological significance of hydrophobicity in maintaining plant fitness, the hierarchical surface architectures such as epidermal roughness and epicuticular wax crystals that determine hydrophobicity, and the molecular genetic pathways that control these structures. Special attention is given to rice and lotus, which exemplify superhydrophobic leaf surfaces and serve as valuable structural and genetic models. We highlight how coordinated regulation of epidermal morphogenesis and wax biosynthesis establishes hydrophobicity, and how its natural variation reflects adaptive optimization to ecological conditions. A mechanistic understanding of these processes not only clarifies the evolutionary strategies of plants but also provides a conceptual and practical foundation for engineering leaf surface wettability to improve crop resilience and sustainable agricultural performance.

## Teaser

This review examines the establishment and regulation of leaf surface hydrophobicity. Its biological significance, hierarchical structural organization, and genetic control are clarified and its adaptive roles and potential applications in crop improvement are discussed.

## Introduction

The plant epidermis forms the interface between the plant body and the external environment, serving as the first line of defense against diverse biotic and abiotic stresses and playing a pivotal role in regulating plant–environment interactions under conditions such as drought and submergence. Among its various properties, hydrophobicity—the ability of the surface to repel water—is one of the most fundamental physical characteristics governing interactions between the epidermis and water.

Plant leaves exhibit a wide range of surface hydrophobicity among species, and such variation is thought to reflect evolutionary optimization to the ecological niche which each plant occupies, enhancing survival and fitness in its specific habitat. Generally, the aerial surfaces of terrestrial plants display relatively high hydrophobicity, which provides protection from various environmental stresses encountered on land. Because optimized hydrophobicity confers multiple physiological and ecological advantages, modifying leaf hydrophobicity may represent a promising target trait in crop improvement, particularly for enhancing performance under specific environmental conditions. However, rational modification requires a mechanistic understanding of how hydrophobicity is established across plant species.

Previous reviews have comprehensively summarized various aspects of leaf surface hydrophobicity, including the diversity of surface architectures (Koch *et al.*, 2008; Koch and Barthlott, 2009; Barthlott *et al.*, 2016, 2017), biomimetic applications of superhydrophobicity (Koch and Barthlott, 2009; Bhushan and Jung, 2011; Latthe *et al.*, 2014; Barthlott *et al.*, 2016, 2017; Jeevahan *et al.*, 2018), and cuticle formation (Post-Beittenmiller, 1996; Kunst and Samuels, 2003, 2009; Bernard and Joubès, 2013; Yeats and Rose, 2013; Lee and Suh, 2015; Jolliffe *et al.*, 2023). Nevertheless, no review has yet integrated these findings from the perspective of controlling or engineering plant hydrophobicity.

In this review, we synthesize current knowledge on (i) the major biological functions of hydrophobicity in plants, (ii) studies examining its structural and genetic bases, and (iii) its diversity and adaptive significance across environments. Finally, we discuss how plants regulate hydrophobicity and propose future directions for its artificial manipulation and potential applications in crop breeding.

## Biological significance of leaf hydrophobicity

The biological significance of leaf hydrophobicity has been interpreted in multiple ways (Koch *et al.*, 2008; Koch and Barthlott, 2009; Barthlott *et al.*, 2016, 2017). Here, we summarize the major proposed functions (Fig. 1).

**Fig. 1.**
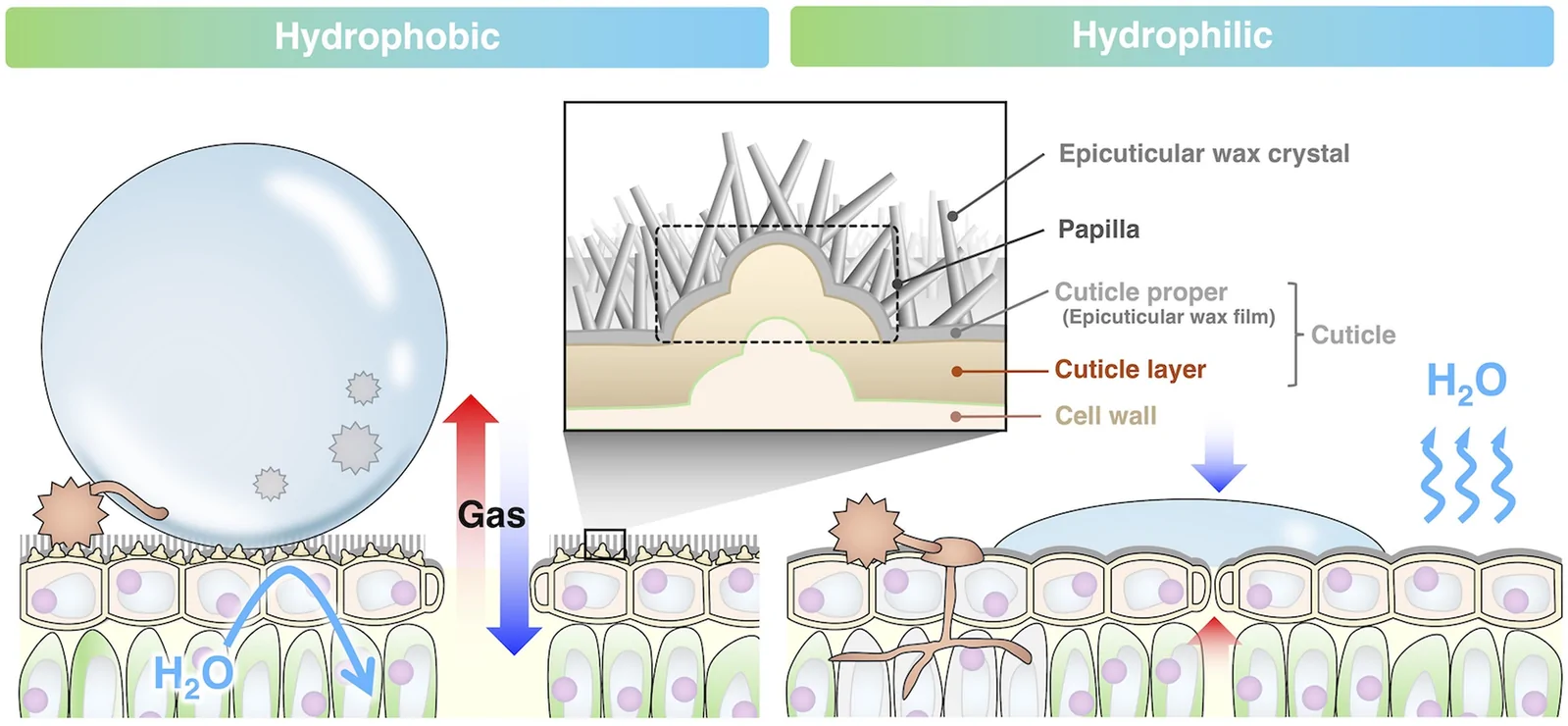
Schematic representation of surface structures and associated functions in hydrophobic and hydrophilic leaves. Left: hydrophobic surface. Water droplets on highly hydrophobic leaves form nearly spherical shapes. Pathogenic microorganisms cannot proliferate on such surfaces and are removed together with droplets via the lotus effect. Gas exchange (arrows) through the stomata remains active, while the cuticular wax layer prevents internal water loss. Right: hydrophilic surface. Water droplets on hydrophilic leaves spread out into thin films. Pathogenic microorganisms can utilize the retained water to proliferate. Stomatal blockage impairs gas exchange (arrows) and reduces photosynthetic efficiency. When the cuticular wax layer is thin, excessive water loss from the leaf interior may occur. Center: a highly hydrophobic leaf surface. The epidermis possesses convex microstructures (papillae) formed through cell morphogenesis and a hierarchically organized array of three-dimensional wax crystals. The epidermal surface is covered by a cuticle composed of hydrophobic compounds, which can be divided into a cutin-rich zone (the cuticle layer) and an overlying wax-rich zone (the cuticle proper or the epicuticular wax).

One of the most important roles of hydrophobicity is protection against pathogens (Fig. 1). Plant surfaces are continuously exposed to diverse pathogenic microorganisms, including bacteria and fungi, most of which require liquid water during at least one stage of their infection cycle (Hamer *et al.*, 1988; Huber and Gillespie, 1992). A hydrophobic surface reduces the likelihood that water droplets carrying microbial propagules adhere to the leaf, thereby limiting pathogen proliferation and decreasing infection risk (Koch *et al.*, 2008; Koch and Barthlott, 2009; Barthlott *et al.*, 2017).

Hydrophobicity also helps maintain essential leaf functions, particularly photosynthesis. When water droplets expand on the leaf surface, photosynthetic efficiency declines (Ishibashi and Terashima, 1995; Ishibashi *et al.*, 1996; Hanba *et al.*, 2004). This reduction arises not only from reversible effects, such as surface water films that block stomatal pores and inhibit gas exchange (Brewer and Smith, 1995) (Fig. 1), but also from irreversible effects that directly impair photosynthetic performance (Ishibashi and Terashima, 1995). Furthermore, stomatal occlusion associated with reduced hydrophobicity limits water-vapor release, thereby disrupting transpiration, temperature regulation, and water transport within the plant (Farquhar and Sharkey, 1982).

Although the effect is indirect, a high degree of hydrophobicity contributes to enhanced tolerance to drought (Koch *et al.*, 2008; Lee and Suh, 2015; Jolliffe *et al.*, 2023) (Fig. 1). Leaf hydrophobicity largely depends on the cuticle, a hydrophobic coating deposited on the outer surface of epidermal cells. Based on chemical composition, the cuticle is generally divided into two regions: a cutin-rich layer (the cuticle layer) and an overlying wax-rich layer (the cuticular proper or epicuticular wax) (Bernard and Joubès, 2013; Yeats and Rose, 2013; Barthlott *et al.*, 2017) (Fig. 1). Epicuticular wax accumulates on the cuticle surface either as a thin film or as crystalline deposits. This wax not only imparts hydrophobicity but also plays a key role in regulating permeability between the plant interior and the external environment (Fernández *et al.*, 2017). Reduced epicuticular wax deposition decreases hydrophobicity and increases cuticular permeability, resulting in excessive water loss under drought conditions (Islam *et al.*, 2009; Li *et al.*, 2019; Yang *et al.*, 2022). Such water loss suppresses growth and, if prolonged, can eventually cause plant death. Indeed, mutants defective in cuticular wax biosynthesis typically display diminished hydrophobicity and reduced drought tolerance (Xue *et al.*, 2017).

Additional functions have been proposed, including regulation of leaf mass after wetting, retention of air layers under water, and altered interactions with insects (Koch *et al.*, 2008; Barthlott *et al.*, 2016, 2017), although quantitative evidence for these roles remains limited. Overall, hydrophobicity should not be regarded as a merely physical attribute; rather, it is an integrated component of plant survival strategies that support physiological and ecological functions such as pathogen defense, maintenance of photosynthesis, and water management.

## Studies on plant hydrophobicity

Research on plant hydrophobicity has mainly focused on the diversity of leaf surface structures and their relationship to wettability (Koch *et al.*, 2008) (Table 1). Since the discovery of the lotus effect (Barthlott and Neinhuis, 1997; Neinhuis and Barthlott, 1997)—the self-cleaning property of extremely water-repellent surfaces exemplified by lotus leaves—numerous studies have explored the artificial fabrication and industrial applications of superhydrophobic surfaces (Koch and Barthlott, 2009; Bhushan and Jung, 2011; Latthe *et al.*, 2014; Barthlott *et al.*, 2016, 2017; Jeevahan *et al.*, 2018). In addition, genetic analyses of mutants with reduced hydrophobicity, as well as comparative studies on the diversity and ecological adaptation of hydrophobic surfaces, have been reported (Table 1).

**Table 1.** Summary of representative studies on leaf surface hydrophobicity in plants

Category	Plant species	Target traits or genes	Key findings or insights related to hydrophobicity	References
**Surface structure**	96 perennial urban plants	Epicuticular wax structure	The type of epicuticular wax structure varied among plant functional types and families. Leaf wettability followed the order: thin film>platelets=crusts>tubules.	Muhammad *et al.* (2020)
	Various plant species	Micro- and nanostructures of the leaf surface	Comprehensive review explaining the relationship between hydrophobicity and leaf surface architecture.	Koch *et al.*, (2008); Koch and Barthlott (2009); Barthlott *et al.* (2016, 2017)
	Eight superhydrophobic species	Epidermal cell and epicuticular wax structures	Superhydrophobic leaves exhibit either a micro–nano dual (binary) structure or a microscale linear (unitary) structure.	Guo and Liu (2007)
	200 plant species with superhydrophobicity	Epidermal cell and epicuticular wax structures	Persistent hydrophobicity is associated with convex to papillate epidermal cells and an extremely dense epicuticular wax layer.	Neinhuis and Barthlott (1997)
	Two rice (*Oryza sativa*) cultivars	Epidermal cell structure and wax content	The cultivar with higher papilla density and greater wax content exhibited stronger hydrophobicity than the one with lower values for these traits.	Ji *et al.* (2021)
**Genetic factors**	*Oryza sativa*	Regulator of cuticular wax biosynthesis (*OsMYB60*)	Mutant leaves show reduced hydrophobicity and wax deposition, resulting in lower desiccation tolerance.	Jian *et al.* (2022)
	*Zea mays*	Regulator of cuticular wax biosynthesis (*ZmEREB46*)	Mutant leaves display decreased wax crystal density and increased droplet adhesion, leading to reduced drought tolerance.	Yang *et al.* (2022)
	*Zea mays*	Regulator of cuticular wax transport (*GLOSSY6*)	Mutant leaves show fewer epicuticular wax crystals and enhanced water droplet adhesion. The causal gene encodes a DUF538-domain protein involved in intracellular wax transport.	Li *et al.* (2019)
	*Oryza sativa*	Cuticular wax biosynthesis (*WSL2–WSL5*)	Mutant leaves exhibit decreased hydrophobicity and wax deposition, accompanied by reduced drought tolerance.	Mao *et al.* (2012); Gan *et al.* (2017); Wang *et al.* (2017); Zhang *et al.* (2020)
	*Oryza sativa*	Cuticular wax biosynthesis (*LGF1*)	The *lgf1* mutant shows reduced hydrophobicity and is unable to form a gas film underwater.	Kurokawa *et al.* (2018)
	*Medicago truncatula*	Regulator of cuticular wax biosynthesis (*IRG1/PALM1*)	Mutant leaves completely lack abaxial wax crystals and show decreased surface hydrophobicity. Loss-of-function mutations alter wax composition and confer resistance to certain biotrophic fungi.	Uppalapati *et al.* (2012)
**Diversity, adaptation, and evolution**	200 plant species with superhydrophobicity	Epidermal cell and epicuticular wax structures	Overview of how hydrophobicity arises across different species and habitats.	Neinhuis and Barthlott (1997)
	96 perennial urban plants	Epidermal cell and epicuticular wax structures	Leaf hydrophobicity decreases over time in many species, with seasonal variation in wax crystal structure.	Muhammad *et al.* (2020)
	Ten peat bog species	Environmental parameters and plant functional traits	Canopy height and leaf longevity correlate with wettability; adaxial hydrophobicity correlates with habitat moisture.	Sikorska *et al.* (2017)
	227 Himalayan species	Environmental parameters	Leaf hydrophobicity decreases with increasing elevation and decreasing temperature.	Aryal and Neuner (2010)
	37 Patagonian species	Leaf epidermal characters and environmental parameters	Although not statistically significant, leaves from drier habitats tended to be less wettable.	Brewer and Nuñez (2007)
	>150 tropical forest species	Leaf morphology and environmental parameters	Leaf hydrophobicity showed no consistent relationship with environmental or phylogenetic factors.	Goldsmith *et al.* (2017)
	41 species representing major plant lineages	Core gene set for cuticle-related pathway (32 genes)	Genetic components of the cuticle first appeared in algae, but the key biosynthetic machinery originated in the last common ancestor of land plants.	Kong *et al.* (2020)

A fundamental aspect of hydrophobicity research is the quantitative evaluation of surface-wetting behavior and the structural identification of surface features by SEM. Comprehensive reviews have described the underlying physical principles and measurement techniques of hydrophobicity (Bhushan and Jung, 2011; Bhushan, 2012; Barthlott *et al.*, 2017; Jeevahan *et al.*, 2018); here, we briefly outline the most basic metric for assessing leaf wettability—the contact angle (CA) (Fig. 2). The static CA is defined as the angle formed at the interface between a liquid droplet and a solid surface (Fig. 2A). Based on CA, leaf wettability is generally classified into four categories (Koch *et al.*, 2008; Koch and Barthlott, 2009; Latthe *et al.*, 2014; Barthlott *et al.*, 2017; Jeevahan *et al.*, 2018): when CA is <10°, the surface is superhydrophilic; 10°≤CA<90° indicates hydrophilicity; 90°≤CA<150° indicates hydrophobicity; and CA ≥150° defines superhydrophobicity (Fig. 2C). On hydrophobic surfaces, water droplets form nearly hemispherical or spherical shapes. When the surface is tilted, the droplet begins to roll at a specific angle—known as the tilt angle (TA)—and the difference between advancing and receding CAs represents hysteresis (Fig. 2B). In superhydrophobic surfaces, a TA <10° and low hysteresis allow droplets to remove dust particles while rolling, producing the characteristic self-cleaning lotus effect (Jeevahan *et al.*, 2018). Particularly for superhydrophobic species such as *Nelumbo nucifera*, extensive research has elucidated the structural, chemical, and physical properties underlying their exceptional water repellency (Ensikat *et al.*, 2011). The following sections summarize representative studies examining the relationships among surface structure, genetics, and ecological adaptation, with emphasis on traits relevant to the modification of hydrophobicity in plants (Table 1).

**Fig. 2.**
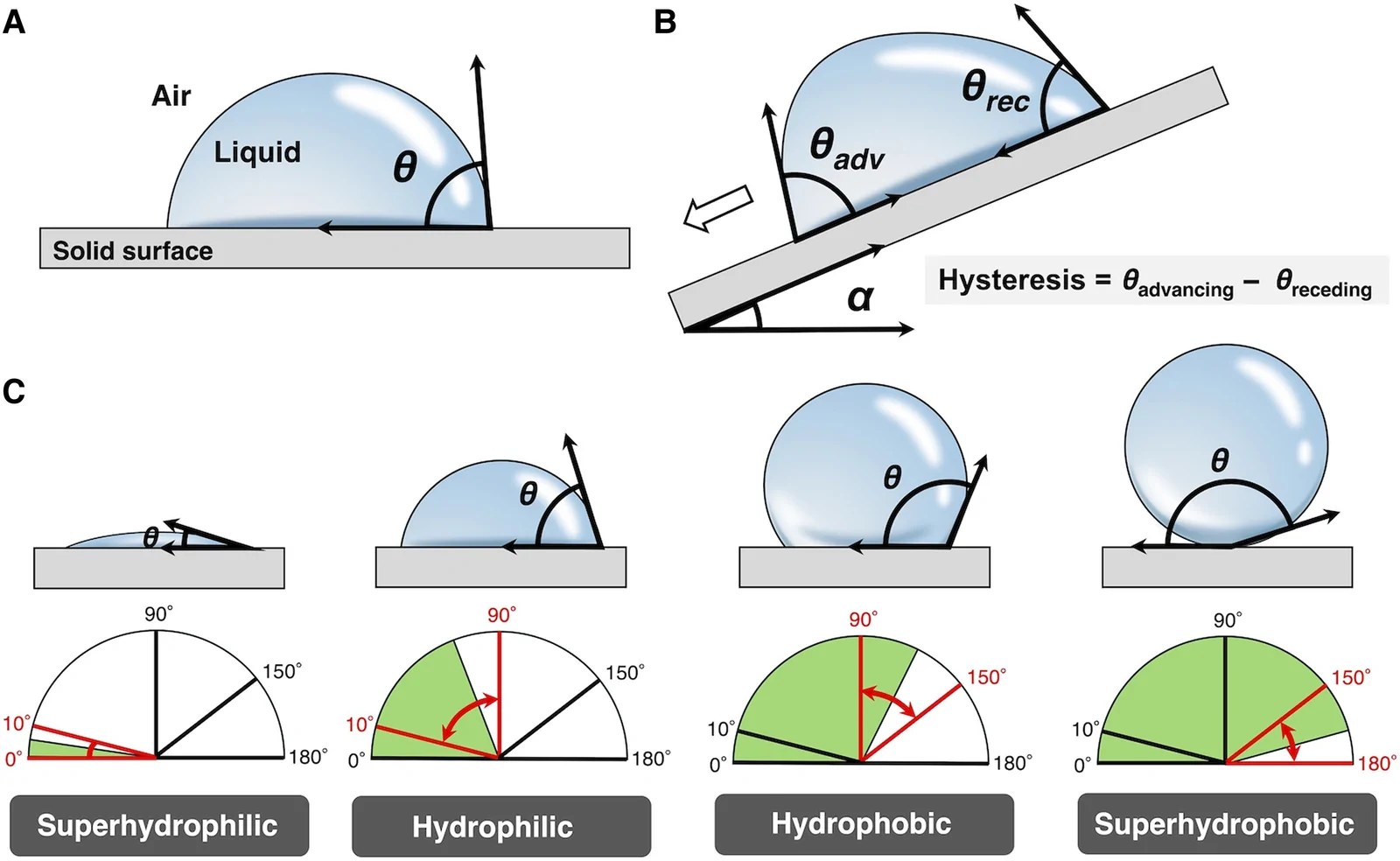
Relationship between contact angle and surface wettability. (A) Definition of the contact angle (θ), measured between the solid surface and the tangent to the liquid–gas interface. (B) When the solid surface supporting a droplet is tilted, the droplet begins to roll at a certain inclination angle—the tilt angle (α). The difference between advancing and receding contact angles (θ_adv_ and θ_rec_) defines the hysteresis, which reflects the adhesive force between the liquid and the surface. (C) Classification of surface wettability based on the contact angle (CA). Surfaces with CA <10° are superhydrophilic; 10°≤CA<90°, hydrophilic; 90°≤CA<150°, hydrophobic; and CA ≥150°, superhydrophobic.

### Structural traits involved in hydrophobicity

Because the CA reflects how a liquid interacts with a solid surface, the plant factors determining hydrophobicity ultimately depend on surface architecture. Although leaf surface morphologies are highly diverse, hydrophobicity is primarily governed by two hierarchical structural levels—micro- and nanoscale features (Fig. 3). Observations of >200 hydrophobic plant species revealed that almost all possessed such hierarchical surfaces (Neinhuis and Barthlott, 1997).

**Fig. 3.**
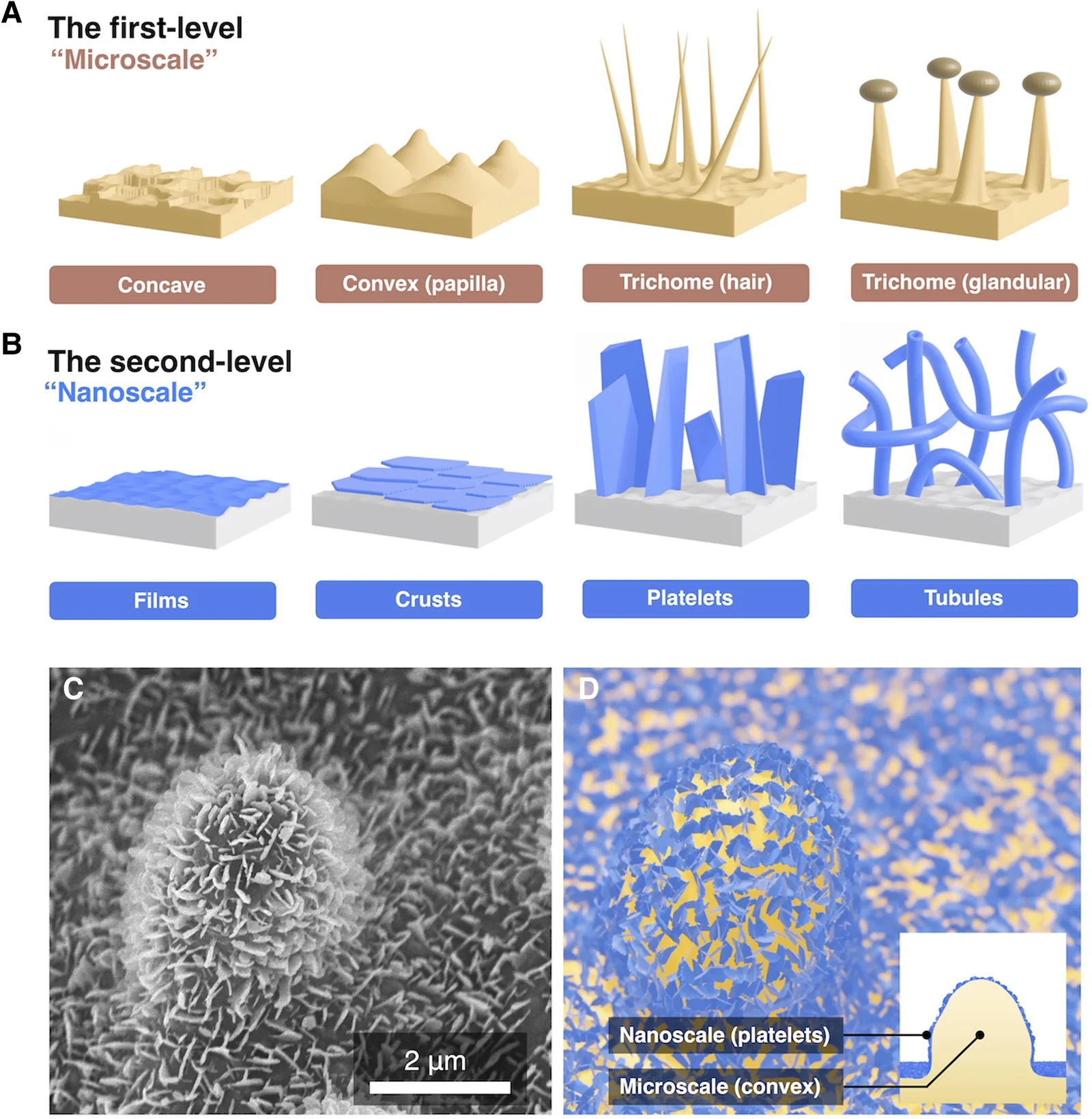
Hierarchical surface structures associated with leaf hydrophobicity. (A) The first-level (microscale) structures (light brown). Epidermal cells may be concave, convex (papillate), or elongated into trichomes—either hair-like or glandular. (B) The second-level (nanoscale) structures (blue). Epicuticular wax crystals may occur as films, crusts, platelets, or tubules. (C) Surface structure of a superhydrophobic rice leaf, showing a hierarchical architecture composed of papillae and platelet-shaped wax crystals. (D) Schematic illustration of a hierarchically structured surface exhibiting superhydrophobicity. Light brown indicates the first-level (microscale) structure, and blue indicates the second-level (nanoscale) structure.

The first-level (microscale) structures consist of cell surface roughness and trichomes (Fig. 3A). These cellular features vary widely, ranging from concave to convex shapes relative to the epidermal layer, and even to hair-like protrusions (Koch *et al.*, 2008). Among these, a well-known structure closely associated with hydrophobicity is the papilla—a microscale protrusion formed through cell morphogenesis, typically occurring singly or in clusters on the epidermal surfaces of superhydrophobic species such as lotus and rice (Guo and Liu, 2007) (Fig. 3C). Trichomes, by contrast, originate from one or several epidermal cells and may be either hair-like or glandular. Their shapes are highly variable and serve multiple functions, and several studies have demonstrated their influence on surface wettability (Koch *et al.*, 2008; Barthlott *et al.*, 2016). Guo and Liu (2007) also found that leaf surfaces completely covered with trichomes exhibit superhydrophobicity. Because the trichomes themselves lack finer substructures, the authors referred to this morphology as a ‘unitary structure’.

The second-level (nanoscale) structures consist of epicuticular waxes secreted onto the epidermal surface (Fig. 3B). The morphology of these crystals ranges from thin two-dimensional films to three-dimensional crusts, platelets, or tubules, and often reflects plant phylogeny (Barthlott *et al.*, 1998) (Fig. 3B). In a survey of 96 urban plant species, Muhammad *et al.* (2020) found that the type of epicuticular wax structure differed among plant functional types and families, with hydrophobicity increasing in the order: thin film<platelet/crust<tubule. Wax microstructure is thought to arise through self-assembly driven by chemical composition (Jeffree *et al.*, 1975; Jetter and Riederer, 1994; Barthlott *et al.*, 1998; Koch and Ensikat, 2008); for example, nonacosan-10-ol isolated from plant waxes can recrystallize into tubular structures *in vitro* under specific conditions (Jetter and Riederer, 1994).

Comparative analyses across various species have classified combinations of these two hierarchical structures in relation to wettability categories (Koch *et al.*, 2008; Barthlott *et al.*, 2017). In superhydrophobic species, three-dimensional wax structures are essential and, in lotus, the combination of such wax crystals with papillate epidermal cells produces nearly perfect superhydrophobicity (Guo and Liu, 2007; Ensikat *et al.*, 2011). Furthermore, an analysis of water droplet deposition in two rice cultivars differing in surface structure revealed that the cultivar with a higher papilla density and greater wax content exhibited stronger hydrophobicity (Ji *et al.*, 2021). Thus, plant surface hydrophobicity is, in most cases, achieved through a hierarchical architecture composed of epidermal microstructures and nanocrystalline epicuticular waxes, and the precise combination of these two layers determines the degree of superhydrophobicity (Fig. 3C, D).

### Genes involved in hydrophobicity

As described above, leaf hydrophobicity depends on both the epidermal microstructure and the nanostructure of wax crystals; therefore, genes associated with these two components are expected to play central roles.

Although numerous genes regulating epidermal cell shape and trichome differentiation have been identified, genes that directly affect hydrophobicity through epidermal morphogenesis remain scarce—largely because few genetic studies have targeted species possessing superhydrophobic surfaces. Rice (*Oryza sativa*), which exhibits papillate epidermal cells resembling those of lotus (Guo and Liu, 2007), serves as an excellent model for such analyses. The *BRIGHT GREEN LEAF* (*BGL*) gene, for example, encodes a ROP guanine nucleotide exchange factor (RopGEF) that activates Rho-family GTPases (Yoo *et al.*, 2011). The *bgl* mutant lacks small papillae on the epidermis, resulting in lighter green leaves, although the direct effect on hydrophobicity was not initially tested. A subsequent comprehensive analysis of rice wetting leaf mutants demonstrated that those lacking papillae showed only a modest (∼10°) decrease in CA, whereas mutants defective in wax accumulation exhibited far greater reductions, in some cases becoming hydrophilic (CA <90°) (Hiraiwa *et al.*, 2024). These results suggest that in rice, the contribution of papillae (first-level structure) to hydrophobicity is smaller than that of wax crystals (second-level structure).

In contrast, numerous genes affecting epicuticular wax biosynthesis are known to influence hydrophobicity. Epicuticular waxes are complex mixtures of hydrophobic compounds derived from very long chain fatty acids (VLCFAs; C26–C34) secreted by epidermal cells (Bernard and Joubès, 2013; Yeats and Rose, 2013; Lee and Suh, 2015). Their compositions vary among species but generally include fatty acids, aldehydes, alcohols, alkanes, ketones, and wax esters. The biosynthesis of these waxes begins with the production of C16 and C18 fatty acids in plastids, which are subsequently activated as long-chain acyl-CoAs in the cytosol. After the process to create these precursors, they are elongated to C20–C34 VLCFA-CoAs by the fatty acid elongase (FAE) complex on the endoplasmic reticulum membrane, comprising β-ketoacyl-CoA synthase (KCS), β-ketoacyl-CoA reductase (KCR), β-hydroxy-acyl-CoA dehydratase (HCD), and enoyl-CoA reductase (ECR) (Batsale *et al.*, 2021). These elongated molecules then enter either the alkane-forming pathway (yielding aldehydes, alkanes, secondary alcohols, and ketones) or the alcohol-forming pathway (yielding primary alcohols and wax esters), after which transporters export the waxes to the cell surface (Kunst and Samuels, 2003; Bernard and Joubès, 2013).

In *Arabidopsis thaliana*, genes within these pathways were largely identified through analyses of *eceriferum* (*cer*) mutants, which display glossy, light-green stems and siliques due to decreased wax deposition (Koornneef *et al.*, 1989; Hannoufa *et al.*, 1993; Jenks *et al.*, 1995). Although *cer* mutants clearly exhibit reduced surface wax crystals, their hydrophobicity has rarely been directly measured. In addition to *cer* mutants, several mutants with altered epidermal wax accumulation have been described, and some of these exhibit organ fusion and other developmental abnormalities (Chen *et al.*, 2003; Kurdyukov *et al.*, 2006). Comparable phenotypes have also been reported in rice (Ito *et al.*, 2011; Tsuda *et al.*, 2013), collectively suggesting that wax biosynthesis contributes not only to the physicochemical properties of plant surfaces but also to fundamental processes of epidermal development. In contrast, *wax crystal-sparse leaf* (*wsl*) mutants in rice have been characterized, in which epicuticular wax crystals are sparsely distributed on the leaf surface and are associated with reduced leaf water repellency (Yu *et al.*, 2008; Mao *et al.*, 2012; Gan *et al.*, 2016, 2017; Zhang *et al.*, 2020). All *WSL* genes characterized to date encode enzymes involved in wax biosynthesis: *WSL1* encodes a KCS catalyzing elongation of C20–C24 VLCFAs (Yu *et al.*, 2008); *WSL4* encodes another KCS responsible for elongation beyond C22 (Gan *et al.*, 2017; Wang *et al.*, 2017); *WSL3* encodes a KCR, another FAE complex component (Gan *et al.*, 2016); *WSL2* affects accumulation of C22–C32 fatty acids (Mao *et al.*, 2012); and *WSL5* participates in primary alcohol formation (Zhang *et al.*, 2020). Additionally, *Leaf Gas Film 1* (*LGF1*) influences the balance between C30 primary alcohols and aldehydes; *lgf1* mutants exhibit a marked drop in CA to the hydrophilic range (CA <90°) (Kurokawa *et al.*, 2018). Transcriptional regulators such as *ZmEREB46* (maize) and *OsMYB60* (rice) modulate wax biosynthetic gene expression and thereby affect hydrophobicity (Jian *et al.*, 2022; Yang *et al.*, 2022). In *Medicago truncatula*, the *IRG1/PALM1* transcription factor gene controls expression of genes for long chain fatty acid synthesis and transport; mutants specifically lose epicuticular waxes on the abaxial leaf surface (Uppalapati *et al.*, 2012). In the *glossy6* (*gl6*) mutant of maize, water droplets remain on the leaf surface after spraying, indicating reduced surface hydrophobicity (Li *et al.*, 2019). Similar phenotypes occur in mutants defective in wax biosynthesis genes; however, in *gl6*, a decrease in wax secretion to the epidermis has been reported. The causal gene encodes a protein containing a DUF538 domain, suggesting its potential involvement in the intracellular transport of cuticular waxes (Li *et al.*, 2019).

Analyses using various mutants illustrate the relationships among the two hierarchical surface structures, hydrophobicity, and associated genes on superhydrophobic leaves (Fig. 4). In the wild type, normal wax-related genes and genes controlling epidermal cell morphogenesis act cooperatively to produce a superhydrophobic surface. When mutations disrupt the genes responsible for epidermal cell patterning—such as *BGL* in rice, which regulates papilla formation—the loss of surface protrusions weakens hydrophobicity, converting superhydrophobicity to hydrophobicity. In contrast, mutations in wax-related genes lead to the loss or malformation of wax crystals and, in severe cases, render the leaf surface hydrophilic. Thus, at least in rice, the formation of epidermal microstructures elevates hydrophobicity to the superhydrophobic level and probably confers additional functional advantages such as self-cleaning ability and flood tolerance.

**Fig. 4.**
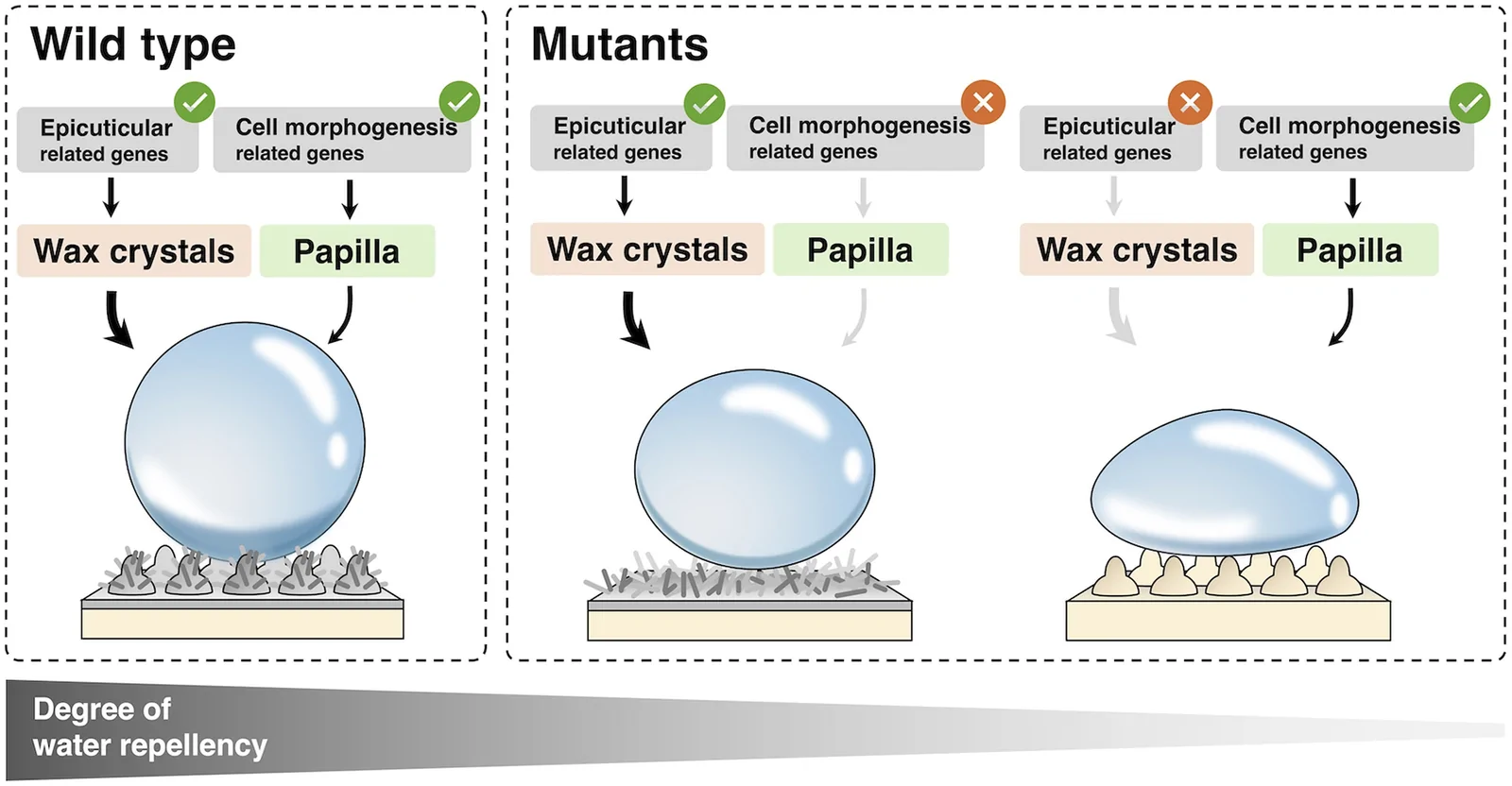
Relationships among hierarchical structures, hydrophobicity, and associated genes on superhydrophobic leaf surfaces. In the wild type, coordinated activity of wax-related genes and genes involved in epidermal cell morphogenesis produces a superhydrophobic leaf surface. When mutations occur in genes controlling cell morphogenesis, the convex epidermal structures (papillae) are lost; likewise, mutations in wax-related genes lead to abnormal wax crystal formation. In both cases, leaf surface hydrophobicity decreases, resulting in hydrophobic or even hydrophilic surfaces.

Overall, leaf hydrophobicity is governed by the concerted activity of genes controlling epidermal morphogenesis and those involved in wax biosynthesis, modification, and transport. Studies in rice are now elucidating this molecular basis in detail.

### Diversity and environmental adaptation of hydrophobicity

Understanding the diversity of hydrophobicity and its ecological relevance is essential for clarifying both its biological significance and the principles of adaptive optimization. One of the most comprehensive analyses is that of Neinhuis and Barthlott (1997), who examined ∼200 plant species for hydrophobicity and ecological traits. They proposed that hydrophobicity levels vary markedly among habitats: forest plants generally showed weak hydrophobicity, particularly in tropical rainforests where wax erosion by heavy rainfall hinders the maintenance of water-repellent surfaces. In contrast, many wetland and aquatic plants exhibited strong hydrophobicity, which functions mainly to prevent pathogen adhesion and infection through surface self-cleaning. In open environments, such as disturbed sites or sandy shores, hydrophobicity helps prevent the deposition of dust or salt, and reduces leaf temperature by limiting water film formation. Thus, hydrophobicity tends to be prominent in herbaceous plants adapted to moist or disturbed habitats, serving crucial ecological roles in self-cleaning and pathogen defense (Neinhuis and Barthlott, 1997). A separate analysis of 96 species revealed that hydrophobicity can vary seasonally, reflecting erosion of wax crystals by air pollution or mechanical stress, as well as activation of defensive responses against fungal pathogens (Muhammad *et al.*, 2020). Studies of 10 species common in peat bogs further showed correlations between wettability and habitat moisture, with more hydrophilic leaves occurring in canopy-forming or long-lived species (Sikorska *et al.*, 2017). Furthermore, measurements of leaf wettability in 227 plant species along altitudinal gradients on both moist and dry slopes of the Nepal Himalayas revealed a marked decrease in wettability with increasing elevation and decreasing temperature. This finding suggests that high hydrophobicity in alpine regions may help reduce plant damage caused by the freezing of water droplets on leaf surfaces (Aryal and Neuner, 2010). Within the genus *Oryza*, screening across diverse genome types demonstrated that semi-aquatic species typically have higher hydrophobicity, whereas shade-adapted forest species show markedly reduced hydrophobicity, suggesting correlations among habitat, genome type, and leaf surface traits (Hiraiwa *et al.*, 2024).

In contrast, an analysis of leaf wettability in 37 species inhabiting steppes, ecotones, and temperate rainforests along a moisture gradient in Argentine Patagonia showed that leaves from drier habitats tended to be less wettable and retain fewer droplets on the leaf surface. However, the overall differences among habitats were not statistically significant (Brewer and Nuñez, 2007). A survey of >150 species across nine tropical forest communities also found no consistent relationship between leaf hydrophobicity and environmental gradients such as rainfall, temperature, or humidity, or with phylogenetic relatedness (Goldsmith *et al.*, 2017). Hydrophobicity is thought to depend not only on interactions with environmental factors but also on intrinsic constraints such as the energetic cost of producing epidermal waxes. Therefore, drawing general conclusions about the relationship between hydrophobicity and environmental adaptation will require a much larger number of diverse and comprehensive studies.

Genetic evidence linking hydrophobicity to adaptive function remains limited. As mentioned in the section on genes involved in hydrophobicity, in rice, *wsl* mutants exhibit increased drought sensitivity (Yu *et al.*, 2008; Mao *et al.*, 2012; Zhang *et al.*, 2020), and in *M. truncatula*, *irg1/palm1* mutants alter spore differentiation of both anthracnose and non-host rust pathogens (Uppalapati *et al.*, 2012). A particularly striking example concerns underwater gas exchange in rice. Because rice grows in flooded fields, its leaves are often partially submerged. Temporary submergence limits gas diffusion and restricts photosynthesis and respiration; however, some aquatic and semi-aquatic plants, including rice, maintain thin gas films on submerged leaf surfaces, enabling underwater gas exchange (Pedersen *et al.*, 2009). In *lgf1* mutants, the loss of surface hydrophobicity prevents gas film retention and severely impairs photosynthesis underwater (Kurokawa *et al.*, 2018). These findings indicate that high leaf hydrophobicity in rice facilitates efficient underwater gas exchange and confers flood tolerance.

Several studies have also explored the relationship between hydrophobicity and environmental adaptation from an evolutionary perspective (Xue *et al.*, 2017; Kong *et al.*, 2020). Kong *et al.* (2020) demonstrated through comparative genomic analyses that a core set of 32 genes involved in cuticle biosynthesis—including those responsible for the synthesis and regulation of epidermal waxes—were already present in the common ancestor of land plants. In addition, comparative analyses of cuticle composition among 13 representative land plant species (ranging from bryophytes to angiosperms) revealed a phylogenetic increase in both cutin and wax content, along with a higher proportion of hydrophobic compounds. Measurements of CAs on leaves or phyllids showed that surface hydrophobicity increased during evolution, reaching its highest level in seed plants. These findings indicate that the quantitative and compositional enhancement of cutin and wax strengthened cuticular hydrophobicity and contributed to improved desiccation tolerance during land plant evolution (Kong *et al.*, 2020). Moreover, nonacosan-10-ol, an aliphatic secondary alcohol that contributes to the superhydrophobicity of lotus and ginkgo leaves, is found exclusively in land plants. The biosynthetic pathway for this compound is therefore thought to have evolved as an adaptation enabling the acquisition of surface hydrophobicity (Barthlott *et al.*, 2016).

Collectively, these studies suggest that hydrophobicity is a flexibly regulated adaptive trait, fine-tuned to environmental conditions and contributing to diverse ecological strategies such as pathogen defense, drought, and flood tolerance.

## Future perspectives

One of the principal goals of studying leaf hydrophobicity is to understand and manipulate the mechanisms that control this property in plants. Although substantial progress has been made, current knowledge remains limited, and several key challenges persist.

A major challenge is to elucidate the morphogenetic mechanisms underlying epidermal structures, particularly the first hierarchical component of the surface—the epidermal cell architecture. In rice, the *BGL* gene, the only gene so far identified to regulate papilla differentiation, encodes a RopGEF that activates Rho-family GTPases (Yoo *et al.*, 2011). Given that this pathway probably involves multiple interacting components—including RopGAPs, RopGTPases, and other regulators (Ou and Yi, 2022; Denninger, 2024)—its detailed characterization will be essential to clarify how papilla number and size are controlled and, consequently, how such microstructures influence surface hydrophobicity. Furthermore, investigating the morphogenetic processes that produce micro-protrusions on epidermal surfaces across diverse superhydrophobic species will enable a comparative understanding of how different lineages have evolved structural strategies for water repellency, opening up new avenues for multi-level manipulation of hydrophobicity.

In contrast, although the chemical and structural diversity of epicuticular waxes is increasingly well characterized, the genetic basis of this diversity remains poorly understood. Wax quantity and composition vary not only among species but also across developmental stages, organs, and even positions within a single leaf (Jetter and Schäffer, 2001; Muhammad *et al.*, 2020; Chen *et al.*, 2023; Zhang *et al.*, 2024). The regulatory mechanisms that modulate wax biosynthetic gene expression at the transcriptional and metabolic levels thus require more in-depth investigation. Addressing these questions will help explain how plants flexibly adjust hydrophobicity to dynamic environmental conditions.

Accordingly, one of the most promising future directions in hydrophobicity research is the genetic dissection of surface structural diversity. Extending molecular genetic approaches beyond model species to encompass a wider range of plants—especially those exhibiting extreme hydrophobicity—will be critical. Advances in genomic resources and genome-editing technologies such as CRISPR/Cas9 now make it feasible to identify and functionally characterize genes that determine surface wettability in both model and non-model species (Hua *et al.*, 2019; Itoh and Sato, 2023; Jolliffe *et al.*, 2023). Such studies will reveal the evolutionary and mechanistic diversity underlying this complex trait.

It is also important to consider how research on hydrophobicity can be translated into practical applications, particularly in crop breeding. Industrial uses of superhydrophobic surfaces are already well established (Lee *et al.*, 2013; Barthlott *et al.*, 2016, 2017; Jeevahan *et al.*, 2018); therefore, this section focuses on agricultural implications. As discussed earlier, high hydrophobicity can confer multiple physiological benefits—reducing pathogen infection risk, maintaining photosynthetic performance, and enhancing drought tolerance (Jolliffe *et al.*, 2023). However, among major crops, only rice possesses naturally superhydrophobic leaves. Introducing superhydrophobicity into other crops could potentially enhance their resilience to both biotic and abiotic stresses via the lotus effect. Because most upland crops are poorly adapted to semi-aquatic environments and highly sensitive to temporary flooding, engineering enhanced hydrophobicity might improve their survival under submergence or high-humidity conditions. Conversely, deliberate reduction of leaf hydrophobicity could also be advantageous in certain contexts—for instance, transiently lowering hydrophobicity may facilitate foliar uptake of agrochemicals or nutrients, thereby contributing to more sustainable and resource-efficient agriculture (Taylor, 2011; Ji *et al.*, 2021).

Future research should therefore aim to establish the molecular foundation for the rational design of hydrophobicity by integrating genetic, transcriptional, and metabolic regulatory frameworks that control papilla (convex epidermal structures) differentiation and wax biosynthesis.

In summary, plant hydrophobicity represents a multi-layered trait shaped by the integrated action of structural, genetic, and ecological factors. Deeper understanding of this complex system will not only cast light on the evolutionary and adaptive strategies of plants but also lay a foundation for innovative applications in crop improvement and biomaterial development.

## References

[erag112-B1] Aryal B, Neuner G. 2010. Leaf wettability decreases along an extreme altitudinal gradient. Oecologia 162, 1–9. 10.1007/s00442-009-1437-3 19727830

[erag112-B2] Barthlott W, Neinhuis C. 1997. Purity of the sacred lotus, or escape from contamination in biological surfaces. Planta 202, 1–8.

[erag112-B3] Barthlott W, Neinhuis C, Cutler D, Ditsch F, Meusel I, Theisen I, Wilhelmi H. 1998. Classification and terminology of plant epicuticular waxes. Botanical Journal of the Linnean Society 126, 237–260.

[erag112-B4] Barthlott W, Mail M, Neinhuis C. 2016. Superhydrophobic hierarchically structured surfaces in biology: evolution, structural principles and biomimetic applications. Philosophical Transactions: Series A, Mathematical, Physical, and Engineering Sciences 374, 20160191. 10.1098/rsta.2016.0191 PMC4928508 27354736

[erag112-B5] Barthlott W, Mail M, Bhushan B, Koch K. 2017. Plant surfaces: structures and functions for biomimetic innovations. Nano-Micro Letters 9, 23. 10.1007/s40820-016-0125-1 PMC6223843 30464998

[erag112-B6] Batsale M, Bahammou D, Fouillen L, Mongrand S, Joubès J, Domergue F. 2021. Biosynthesis and functions of very-long-chain fatty acids in the responses of plants to abiotic and biotic stresses. Cells 10, 1284. 10.3390/cells10061284 PMC8224384 34064239

[erag112-B7] Bernard A, Joubès J. 2013. Arabidopsis cuticular waxes: advances in synthesis, export and regulation. Progress in Lipid Research 52, 110–129. 10.1016/j.plipres.2012.10.002 23103356

[erag112-B8] Bhushan B, Jung YC. 2011. Natural and biomimetic artificial surfaces for superhydrophobicity, self-cleaning, low adhesion, and drag reduction. Progress in Materials Science 56, 1–108.

[erag112-B9] Bhushan B . 2012. Bioinspired structured surfaces. Langmuir 28, 1698–1714. 10.1021/la2043729 22233136

[erag112-B10] Brewer CA, Smith WK. 1995. Leaf surface wetness and gas exchange in the pond lily *Nuphar polysepalum* (Nymphaeaceae). American Journal of Botany 82, 1271–1277.

[erag112-B11] Brewer CA, Nuñez CI. 2007. Patterns of leaf wettability along an extreme moisture gradient in western Patagonia, Argentina. International Journal of Plant Sciences 168, 555–562.

[erag112-B12] Chen JY, Kuruparan A, Zamani-Babgohari M, Gonzales-Vigil E. 2023. Dynamic changes to the plant cuticle include the production of volatile cuticular wax-derived compounds. Proceedings of the National Academy of Sciences, USA 120, e2307012120. 10.1073/pnas.2307012120 PMC10710056 38019866

[erag112-B13] Chen X, Goodwin SM, Boroff VL, Liu X, Jenks MA. 2003. Cloning and characterization of the *WAX2* gene of Arabidopsis involved in cuticle membrane and wax production. The Plant Cell 15, 1170–1185. 10.1105/tpc.010926 PMC153724 12724542

[erag112-B14] Denninger P . 2024. RHO OF PLANTS signalling and the activating ROP GUANINE NUCLEOTIDE EXCHANGE FACTORS: specificity in cellular signal transduction in plants. Journal of Experimental Botany 75, 3685–3699. 10.1093/jxb/erae196 PMC11194304 38683617

[erag112-B15] Ensikat HJ, Ditsche-Kuru P, Neinhuis C, Barthlott W. 2011. Superhydrophobicity in perfection: the outstanding properties of the lotus leaf. Beilstein Journal of Nanotechnology 2, 152–161. 10.3762/bjnano.2.19 PMC3148040 21977427

[erag112-B16] Farquhar GD, Sharkey TD. 1982. Stomatal conductance and photosynthesis. Annual Review of Plant Physiology 33, 317–345.

[erag112-B17] Fernández V, Bahamonde HA, Javier Peguero-Pina J, Gil-Pelegrín E, Sancho-Knapik D, Gil L, Goldbach HE, Eichert T. 2017. Physico-chemical properties of plant cuticles and their functional and ecological significance. Journal of Experimental Botany 68, 5293–5306. 10.1093/jxb/erx302 28992247

[erag112-B18] Gan L, Wang X, Cheng Z, et al 2016. *Wax crystal-sparse leaf 3* encoding a β-ketoacyl-CoA reductase is involved in cuticular wax biosynthesis in rice. Plant Cell Reports 35, 1687–1698. 10.1007/s00299-016-1983-1 27106031

[erag112-B19] Gan L, Zhu S, Zhao Z, et al 2017. *Wax crystal-sparse leaf 4*, encoding a β-ketoacyl-coenzyme A synthase 6, is involved in rice cuticular wax accumulation. Plant Cell Reports 36, 1655–1666. 10.1007/s00299-017-2181-5 28733852

[erag112-B20] Goldsmith GR, Bentley LP, Shenkin A, et al 2017. Variation in leaf wettability traits along a tropical montane elevation gradient. New Phytologist 214, 989–1001. 10.1111/nph.14121 PMC5412938 27463359

[erag112-B21] Guo Z, Liu W. 2007. Biomimic from the superhydrophobic plant leaves in nature: binary structure and unitary structure. Plant Science 172, 1103–1112.

[erag112-B22] Hamer JE, Howard RJ, Chumley FG, Valent B. 1988. A mechanism for surface attachment in spores of a plant pathogenic fungus. Science 239, 288–290. 10.1126/science.239.4837.288 17769992

[erag112-B23] Hanba YT, Moriya A, Kimura K. 2004. Effect of leaf surface wetness and wettability on photosynthesis in bean and pea. Plant, Cell & Environment 27, 413–421.

[erag112-B24] Hannoufa A, McNevin J, Lemieux B. 1993. Epicuticular waxes of *eceriferum* mutants of *Arabidopsis thaliana*. Phytochemistry 33, 851–855.

[erag112-B25] Hiraiwa A, Aiga S, Zhu T, Mimura M, Yoshikawa T, Sato Y, Itoh J-I. 2024. Structural, genetic, and adaptive basis of superhydrophobicity in rice leaves. bioRxiv. doi: 10.1101/2024.12.05.626993. [Preprint]. PMC13413574 42489112

[erag112-B26] Hua K, Zhang J, Botella JR, Ma C, Kong F, Liu B, Zhu J-K. 2019. Perspectives on the application of genome-editing technologies in crop breeding. Molecular Plant 12, 1047–1059. 10.1016/j.molp.2019.06.009 31260812

[erag112-B27] Huber L, Gillespie TJ. 1992. Modeling leaf wetness in relation to plant disease epidemiology. Annual Review of Phytopathology 30, 553–577.

[erag112-B28] Ishibashi M, Terashima I. 1995. Effects of continuous leaf wetness on photosynthesis: adverse aspects of rainfall. Plant, Cell & Environment 18, 431–438.

[erag112-B29] Ishibashi M, Usuda H, Terashima I. 1996. The loss of ribulose-1,5-bisphosphate carboxylase/oxygenase caused by 24-hour rain treatment fully explains the decrease in the photosynthetic rate in bean leaves. Plant Physiology 111, 635–640. 10.1104/pp.111.2.635 PMC157876 12226316

[erag112-B30] Islam MA, Du H, Ning J, Ye H, Xiong L. 2009. Characterization of *Glossy1*-homologous genes in rice involved in leaf wax accumulation and drought resistance. Plant Molecular Biology 70, 443–456. 10.1007/s11103-009-9483-0 19322663

[erag112-B31] Ito Y, Kimura F, Hirakata K, Tsuda K, Takasugi T, Eiguchi M, Nakagawa K, Kurata N. 2011. Fatty acid elongase is required for shoot development in rice. The Plant Journal 66, 680–688. 10.1111/j.1365-313X.2011.04530.x 21309865

[erag112-B32] Itoh J-I, Sato Y. 2023. Understanding plant development for plant breeding. Breeding Science 73, 1–2. 10.1270/jsbbs.73.1 PMC10165340 37168812

[erag112-B33] Jeevahan J, Chandrasekaran M, Britto Joseph G, Durairaj RB, Mageshwaran G. 2018. Superhydrophobic surfaces: a review on fundamentals, applications, and challenges. Journal of Coatings Technology and Research 15, 231–250.

[erag112-B34] Jeffree CE, Baker EA, Holloway PJ. 1975. Ultrastructure and recrystallization of plant epicuticular waxes. New Phytologist 75, 539–549.

[erag112-B35] Jenks MA, Tuttle HA, Eigenbrode SD, Feldmann KA. 1995. Leaf epicuticular waxes of the *eceriferum* mutants in Arabidopsis. Plant Physiology 108, 369–377. 10.1104/pp.108.1.369 PMC157343 12228482

[erag112-B36] Jetter R, Riederer M. 1994. Epicuticular crystals of nonacosan-10-ol: in-vitro reconstitution and factors influencing crystal habits. Planta 195, 257–270.

[erag112-B37] Jetter R, Schäffer S. 2001. Chemical composition of the *Prunus laurocerasus* leaf surface. Dynamic changes of the epicuticular wax film during leaf development. Plant Physiology 126, 1725–1737. 10.1104/pp.126.4.1725 PMC117171 11500570

[erag112-B38] Ji G, Chen H, Zhang Y, Xiang J, Wang Y, Wang Z, Zhu D, Zhang Y. 2021. Leaf surface characteristics affect the deposition and distribution of droplets in rice (*Oryza sativa* L.). Scientific Reports 11, 17846. 10.1038/s41598-021-97061-5 PMC8426486 34497313

[erag112-B39] Jian L, Kang K, Choi Y, Suh MC, Paek N-C. 2022. Mutation of *OsMYB60* reduces rice resilience to drought stress by attenuating cuticular wax biosynthesis. The Plant Journal 112, 339–351. 10.1111/tpj.15947 35984735

[erag112-B40] Jolliffe JB, Pilati S, Moser C, Lashbrooke JG. 2023. Beyond skin-deep: targeting the plant surface for crop improvement. Journal of Experimental Botany 74, 6468–6486. 10.1093/jxb/erad321 PMC10662250 37589495

[erag112-B41] Koch K, Bhushan B, Barthlott W. 2008. Diversity of structure, morphology and wetting of plant surfaces. Soft Matter 4, 1943–1963.

[erag112-B42] Koch K, Ensikat H-J. 2008. The hydrophobic coatings of plant surfaces: epicuticular wax crystals and their morphologies, crystallinity and molecular self-assembly. Micron 39, 759–772. 10.1016/j.micron.2007.11.010 18187332

[erag112-B43] Koch K, Barthlott W. 2009. Superhydrophobic and superhydrophilic plant surfaces: an inspiration for biomimetic materials. Philosophical Transactions of the Royal Society A: Mathematical, Physical, and Engineering Sciences 367, 1487–1509. 10.1098/rsta.2009.0022 19324720

[erag112-B44] Kong L, Liu Y, Zhi P, Wang X, Xu B, Gong Z, Chang C. 2020. Origins and evolution of cuticle biosynthetic machinery in land plants. Plant Physiology 184, 1998–2010. 10.1104/pp.20.00913 PMC7723097 32934149

[erag112-B45] Koornneef M, Hanhart CJ, Thiel F. 1989. A genetic and phenotypic description of *eceriferum* (*cer*) mutants in *Arabidopsis thaliana*. Journal of Heredity 80, 118–122.

[erag112-B46] Kunst L, Samuels AL. 2003. Biosynthesis and secretion of plant cuticular wax. Progress in Lipid Research 42, 51–80. 10.1016/s0163-7827(02)00045-0 12467640

[erag112-B47] Kunst L, Samuels L. 2009. Plant cuticles shine: advances in wax biosynthesis and export. Current Opinion in Plant Biology 12, 721–727. 10.1016/j.pbi.2009.09.009 19864175

[erag112-B48] Kurdyukov S, Faust A, Nawrath C, et al 2006. The epidermis-specific extracellular BODYGUARD controls cuticle development and morphogenesis in Arabidopsis. The Plant Cell 18, 321–339. 10.1105/tpc.105.036079 PMC1356542 16415209

[erag112-B49] Kurokawa Y, Nagai K, Huan PD, et al 2018. Rice leaf hydrophobicity and gas films are conferred by a wax synthesis gene (*LGF1*) and contribute to flood tolerance. New Phytologist 218, 1558–1569. 10.1111/nph.15070 29498045

[erag112-B50] Latthe SS, Terashima C, Nakata K, Fujishima A. 2014. Superhydrophobic surfaces developed by mimicking hierarchical surface morphology of lotus leaf. Molecules 19, 4256–4283. 10.3390/molecules19044256 PMC6270765 24714190

[erag112-B51] Lee SB, Suh MC. 2015. Advances in the understanding of cuticular waxes in *Arabidopsis thaliana* and crop species. Plant Cell Reports 34, 557–572. 10.1007/s00299-015-1772-2 25693495

[erag112-B52] Lee SG, Lim HS, Lee DY, Kwak D, Cho K. 2013. Tunable anisotropic wettability of rice leaf-like wavy surfaces. Advanced Functional Materials 23, 547–553.

[erag112-B53] Li L, Du Y, He C, et al 2019. Maize *glossy6* is involved in cuticular wax deposition and drought tolerance. Journal of Experimental Botany 70, 3089–3099. 10.1093/jxb/erz131 PMC6598097 30919902

[erag112-B54] Mao B, Cheng Z, Lei C, et al 2012. Wax crystal-sparse leaf2, a rice homologue of WAX2/GL1, is involved in synthesis of leaf cuticular wax. Planta 235, 39–52. 10.1007/s00425-011-1481-1 21809091

[erag112-B55] Muhammad S, Wuyts K, Nuyts G, De Wael K, Samson R. 2020. Characterization of epicuticular wax structures on leaves of urban plant species and its association with leaf wettability. Urban Forestry & Urban Greening 47, 126557.

[erag112-B56] Neinhuis C, Barthlott W. 1997. Characterization and distribution of water-repellent, self-cleaning plant surfaces. Annals of Botany 79, 667–677.

[erag112-B57] Ou H, Yi P. 2022. ROP GTPase-dependent polarity establishment during tip growth in plants. New Phytologist 236, 49–57. 10.1111/nph.18373 35832004

[erag112-B58] Pedersen O, Rich SM, Colmer TD. 2009. Surviving floods: leaf gas films improve O_2_ and CO_2_ exchange, root aeration, and growth of completely submerged rice. The Plant Journal 58, 147–156. 10.1111/j.1365-313X.2008.03769.x 19077169

[erag112-B59] Post-Beittenmiller D . 1996. Biochemistry and molecular biology of wax production in plants. Annual Review of Plant Physiology and Plant Molecular Biology 47, 405–430. 10.1146/annurev.arplant.47.1.405 15012295

[erag112-B60] Sikorska D, Papierowska E, Szatyłowicz J, Sikorski P, Suprun K, Hopkins RJ. 2017. Variation in leaf surface hydrophobicity of wetland plants: the role of plant traits in water retention. Wetlands 37, 997–1002.

[erag112-B61] Taylor P . 2011. The wetting of leaf surfaces. Current Opinion in Colloid & Interface Science 16, 326–334.

[erag112-B62] Tsuda K, Akiba T, Kimura F, Ishibashi M, Moriya C, Nakagawa K, Kurata N, Ito Y. 2013. ONION2 fatty acid elongase is required for shoot development in rice. Plant & Cell Physiology 54, 209–217. 10.1093/pcp/pcs169 PMC3583024 23220821

[erag112-B63] Uppalapati SR, Ishiga Y, Doraiswamy V, et al 2012. Loss of abaxial leaf epicuticular wax in *Medicago truncatula irg1/palm1* mutants results in reduced spore differentiation of anthracnose and nonhost rust pathogens. The Plant Cell 24, 353–370. 10.1105/tpc.111.093104 PMC3289574 22294617

[erag112-B64] Wang X, Guan Y, Zhang D, Dong X, Tian L, Qu LQ. 2017. A β-ketoacyl-CoA synthase is involved in rice leaf cuticular wax synthesis and requires a CER2-LIKE protein as a cofactor. Plant Physiology 173, 944–955. 10.1104/pp.16.01527 PMC5291035 27913740

[erag112-B65] Xue D, Zhang X, Lu X, Chen G, Chen Z-H. 2017. Molecular and evolutionary mechanisms of cuticular wax for plant drought tolerance. Frontiers in Plant Science 8, 621. 10.3389/fpls.2017.00621 PMC5408081 28503179

[erag112-B66] Yang Y, Shi J, Chen L, Xiao W, Yu J. 2022. ZmEREB46, a maize ortholog of Arabidopsis WAX INDUCER1/SHINE1, is involved in the biosynthesis of leaf epicuticular very-long-chain waxes and drought tolerance. Plant Science 321, 111256. 10.1016/j.plantsci.2022.111256 35696901

[erag112-B67] Yeats TH, Rose JKC. 2013. The formation and function of plant cuticles. Plant Physiology 163, 5–20. 10.1104/pp.113.222737 PMC3762664 23893170

[erag112-B68] Yoo J-H, Park J-H, Cho S-H, Yoo S-C, Li J, Zhang H, Kim K-S, Koh H-J, Paek N-C. 2011. The rice *bright green leaf* (*bgl*) locus encodes OsRopGEF10, which activates the development of small cuticular papillae on leaf surfaces. Plant Molecular Biology 77, 631–641. 10.1007/s11103-011-9839-0 22038138

[erag112-B69] Yu D, Ranathunge K, Huang H, Pei Z, Franke R, Schreiber L, He C. 2008. *Wax crystal-sparse Leaf1* encodes a beta-ketoacyl CoA synthase involved in biosynthesis of cuticular waxes on rice leaf. Planta 228, 675–685. 10.1007/s00425-008-0770-9 18574592

[erag112-B70] Zhang D, Yang H, Wang X, Qiu Y, Tian L, Qi X, Qu LQ. 2020. Cytochrome P450 family member CYP96B5 hydroxylates alkanes to primary alcohols and is involved in rice leaf cuticular wax synthesis. New Phytologist 225, 2094–2107. 10.1111/nph.16267 31618451

[erag112-B71] Zhang Z, Mistry D, Jetter R. 2024. Micromorphological and chemical characterization of *Drimys winteri* leaf surfaces: the secondary alcohols forming epicuticular wax crystals are accompanied by alkanediol, alkanetriol and ketol derivatives. Plant & Cell Physiology 65, 1245–1260. 10.1093/pcp/pcae053 PMC11369817 38757823

